# Efficacy of bronchial artery embolization in hemoptysis: longitudinal studyon survival and recurrence

**DOI:** 10.1186/s42155-025-00627-x

**Published:** 2025-12-13

**Authors:** Mohammad Sadegh Keshmiri, Bahamin Astani, Fatemeh Sadat Hosseini-Baharanchi, Babak Sharif-Kashani, Mahdi Ahmadinia, Sheida Mohammadi, Sepideh Ranjbar, Leila Saliminejad, Shadi Shafaghi

**Affiliations:** 1 Lung Transplantation Research Center, National Research Institute of Tuberculosis and Lung Diseases (NRITLD), Shahid Beheshti University of Medical Sciences, Tehran, Iran; 2 Department of Biostatistics, School of Public Health, Iran University of Medical Sciences, Tehran, Iran

**Keywords:** Bronchial artery embolization, Massive hemoptysis, Non-massive hemoptysis, Recurrence, Survival, Efficacy

## Abstract

**Background and objectives:**

Managing hemoptysis can be challenging due to recurrences after different treatment methods. This study aimed to assess the efficacy and long-term outcomes of bronchial artery embolization (BAE) in controlling hemoptysis and improving patient survival.

**Methods:**

In this prospective cohort study, patients with hemoptysis undergoing BAE between August 2017 and August 2022 were enrolled and prospectively followed. Clinical characteristics, underlying etiologies, complications, and post-procedural recurrences were prospectively recorded during a 1- to 4-year follow-up period. The survival for each factor was graphed in subgroups by the Kaplan–Meier (KM) curve and presented the estimation of the hazard ratio (HR) with 95% confidence interval (CI) from the univariate Cox proportional hazard (PH) model.

**Results:**

A total of 297 patients (32% female, 58% with massive hemoptysis) were included. The mean survival time (MST) for all-cause mortality was 35.9 months (95% *CI* 33.6–38.3) and for hemoptysis-related death was 45.1 months (95% *CI* 43.8–46.4). The overall recurrence rate was 14.6% at 1-month post-BAE, decreased to 5.7% by the 9th month, and rose again to approximately 20% during long-term follow-up. Recurrence was significantly 71% higher in patients with non-massive hemoptysis (46.6%) compared with those with massive hemoptysis (33.8%, *OR* = 1.71, 95% *CI* 1.05–2.80, *P* = 0.03).

**Conclusions:**

BAE effectively controls life-threatening hemoptysis with favorable long-term survival and acceptable recurrence rates. Optimized management of the underlying pulmonary disease may further improve BAE outcomes and reduce recurrence risk.

**Supplementary Information:**

The online version contains supplementary material available at 10.1186/s42155-025-00627-x.

## Introduction

Hemoptysis is defined as expectoration of blood or bloody sputum from the lower respiratory tract [1]. Common etiologies of hemoptysis are bronchiectasis, bronchial carcinoma and metastases, cystic fibrosis, and respiratory infections such as TB and covid-19 in both acute phase and prolonged covid-19 [2].

Hemoptysis is commonly classified as non-massive (< 200 mL in 24 h) and massive (> 200 mL in 24 h), although the exact threshold for “massive” hemoptysis may vary among studies and institutions [3]. Massive hemoptysis causes asphyxia which is the main reason for mortality and morbidity which could be up to 80% in these patients; therefore, it is essential to treat hemoptysis promptly, and there are multiple therapeutic approaches in this regard [4, 5].

Treatment approaches could be performed in combination with one another; approaches such as using vasoactive drugs are only used for the management of mild to moderate hemoptysis [6]. On the other hand, the hemostatic effect of bronchoscopy as another therapeutic approach usually lasts for a short time [7]. BAE is used as a minimally invasive treatment for hemoptysis introduced by Remy et al. [8]. Previous studies have shown an increased success and efficacy rate of this procedure during the recent years, especially for the management of massive hemoptysis [7, 9].

There is a lack of evidence comparing the short- and long-term efficacy of bronchial artery embolization across different etiologies of hemoptysis. This study aimed to evaluate the success rate of BAE and short- and long-term survival and recurrence rate of hemoptysis among patients with both massive and non-massive hemoptysis caused by different etiologies.

## Material and method

### Study population

In this prospective cohort study, patients with hemoptysis who were undergoing BAE at Masih Daneshvari Hospital between August 2017 and August 2022 were prospectively enrolled and followed for outcomes. This study was approved by the Iranian National Committee for Ethics in Biomedical Research (ethic code: IR.SBMU.NRITLD.REC.1402.006) and followed the ethical guidelines of the Declaration of Helsinki. As this was a prospective observational study, a clinical trial registration number was not required. Also, all participants provided written informed consent before their inclusion in the study. Consent was obtained from each participant to use their clinical data for research purposes, and all participants were made aware of the study’s objectives, procedures, potential risks, and benefits.

Inclusion criteria were patients above 15 years old with hemoptysis who were candidates for BAE. In patients with non-massive hemoptysis, BAE was reserved for those with recurrent or persistent bleeding despite conservative treatment, high-risk clinical features (significant hemoglobin drop or transfusion requirement, cardiopulmonary comorbidities, or nonreversible anticoagulation), or when imaging or angiography suggested a treatable vascular abnormality. Patients with massive hemoptysis were treated more urgently and comprehensively, targeting all visible abnormal feeders during the procedure. Exclusion criteria were patients with high creatinine levels and renal failure, uncorrectable coagulopathy and severe contrast allergy, and patients with hemoptysis who were BAE candidates but whose procedure was unsuccessful due to a lack of abnormal vessels in angiography. The technical success was defined as the ability to embolize the abnormal and hemorrhagic vessels. Before proceeding with BAE, CT scan examination was performed for all patients to identify the extent of disease and localize the possible bleeding site. All patients underwent pre-procedural chest CT scans (non-contrast or contrast-enhanced as clinically indicated) to identify underlying pulmonary pathology. Dedicated CT angiography (CTA) was not routinely performed, and the definitive evaluation of bronchial artery anatomy was achieved through conventional angiography during the BAE procedure.

In order to embolize the bleeding vessels, arterial access was obtained through the right femoral artery. Aortography was performed by inserting the 5 F pigtail catheter (Cordis Co., Vaughan, CA, USA) into the descending aorta. In most of the aortograms, bronchial and intercostal arteries were observed and used as guides to find abnormal blood vessels (torturous, aneurysm, fistula, and ectasia). And if these vessels were not visible, an attempt was made to find abnormal arteries through an investigative approach. Then, using the 5 F C1 (Cobra) catheter (Merit Medical Systems, Inc., South Jordan, UT, USA) or the Tiger 5 F catheter (Merit Medical Systems, Inc.), angiography of the bronchial and intercostal arteries was performed selectively, and in case of abnormalities such as vascular ectasia, vascular torsion, fistula, or hypervascularity, embolization was performed selectively using polyvinyl alcohol (PVA) particles (Contour™, Boston Scientific, USA; size 500–710 µm) [5].

Although this was a prospective study, a sample size estimation was performed to confirm the adequacy of the available data for statistical analysis. For the priory sample size calculation, type I error and type II errors were considered 20% and 5%, respectively. Using *N*_ev_ = ((*Z*_1-α/2_ + *Z*_1-β_)(θ + 1)/(θ−1))^2^ in which *θ* is the hazard ratio [10], *θ* was assumed 1.83 based on a pilot study: the hazard ratio of hemoptysis in men to women in this study. The predicted recurrence rate of hemoptysis was considered 28% [1], and then *N*_total_ = *N*_ev_/*P*_ev_ = 326.

### Data collection and follow-up

**Fig. 1:**
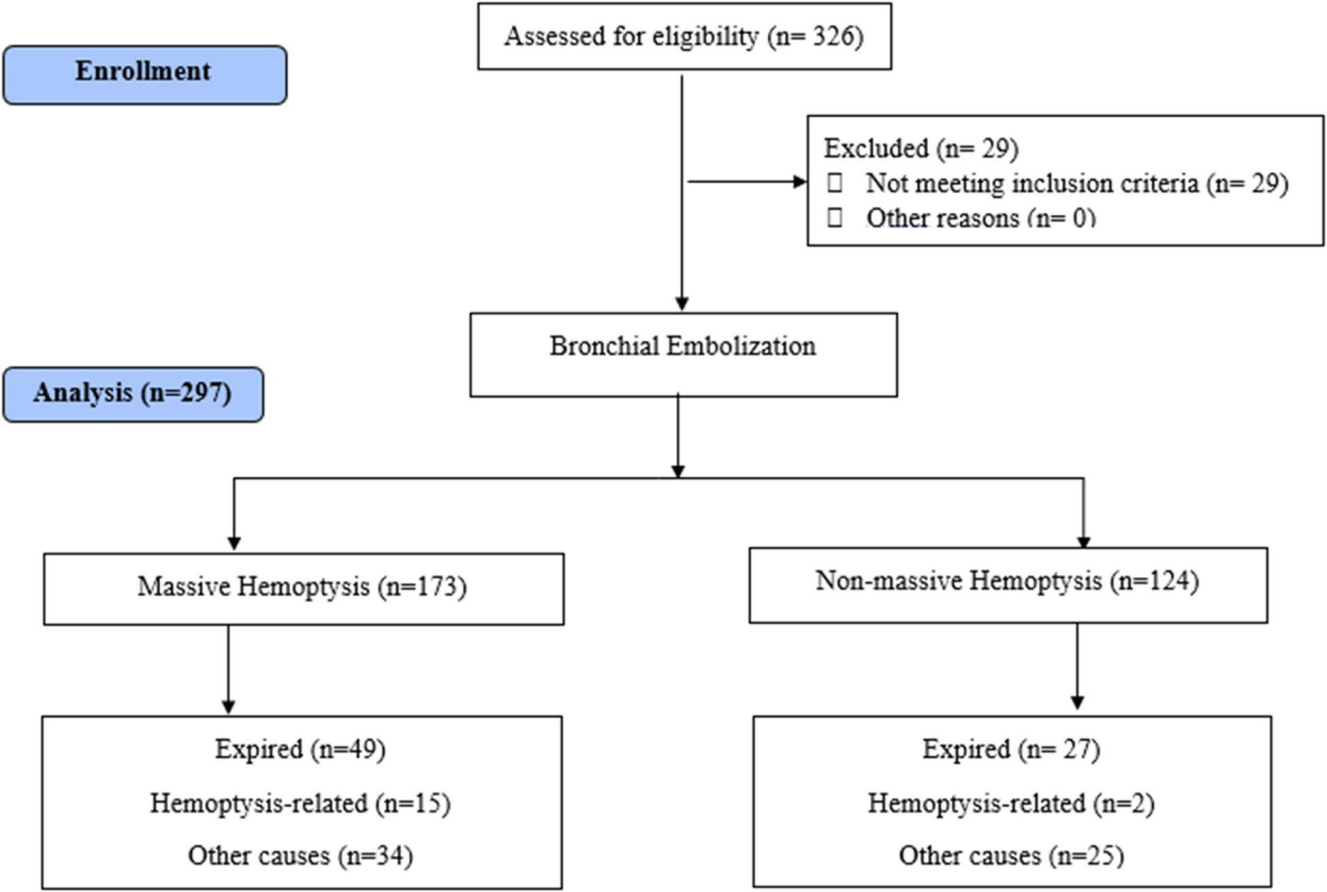
The schematic of the patients’ recruitment

Patient data including age, sex, underlying diseases, severity of hemoptysis, number and side of bleeding vessels, CT scan findings, and procedural details were prospectively recorded at the time of BAE and during follow-up visits. The patient’s general condition, survival, cause of death, hemoptysis recurrence, and treatment procedures used at the time of recurrence were obtained via phone contact, and their data was completed. The patient enrollment process for this study is depicted in Fig. 1, the patient recruitment scheme. Patients’ hemoptysis recurrence and survival were studied in the short and long term. Short-term efficacy was considered as 1 month to 1-year post-BAE, and long term was identified as 1 year to 4 years, annually.

### Statistical analysis

Continuous and categorical data were reported by mean ± standard deviation (SD) and frequency (%), respectively. Survival rates and mean survival time (MST) were reported for all-cause survival and hemoptysis-related survival. Kaplan–Meier (KM) estimation is presented for each factor in addition to HR and 95% CI through Cox PH model. Odds ratio (95%CI) was reported to assess the effect size of different factors on recurrence odds using univariate logistic regression. Kappa measure of agreement was used to assess the concordance between CT scan findings and the side of bleeding vessel embolization. The association between recurrence and death and hemoptysis severity was evaluated by chi-square test. Statistical analysis was conducted using R-studio (4.3.0) considering the level of significance of 0.05.

## Results

Three hundred and 26 patients were referred to the angiographic department for BAE of whom 29 (8.8%) patients were excluded from analysis and follow-up due to a lack of abnormal arteries during the procedure and an inability to perform embolization. A total of 297 patients with hemoptysis undergoing bronchial artery embolization were included in the analysis of whom 173 (58.2%) cases suffered from massive hemoptysis. The mean ± SD age was 54.85 ± 16.58 of whom 95 (32%) were female. Table 1 demonstrates demographic and clinical characteristics, and Table 1S illustrates the embolization and mortality factors. Bilateral abnormalities in CT scans were found in 137 (46.1%) patients. The kappa measure showed agreement of 0.213 between abnormal vessels in CT scan findings and BAE (*P* < 0.001, Table 2S).

**Table 1:** Demographic and clinical characteristics

**Factor**	**Subgroup**	***N* (%)**
Age		
	≤ 30	26 (8.8)
	30–60	135 (45.5)
	≥ 60	136 (45.7)
Gender		
	Female	95 (32.0)
	Male	202 (68.0)
Hemoptysis severity		
	Massive	173 (58.2)
	Non-massive	124 (41.8)
Underlying disease		
	Bronchiectasis	82 (27.6)
	Cancer	49 (16.5)
	Non-TB Infections^*^	33 (11.1)
	Old TB	95 (32.0)
	Others^**^	38 (12.8)
CT scan abnormality side		
	Bilateral disseminated	137 (46.1)
	Right	66 (22.2)
	Left	41 (13.8)
	Normal	53 (17.8)
Embolization frequency		
	1	250 (84.2)
	2	26 (8.7)
	≥ 3	21 (7.1)

^*^Non-TB infections include acute covid-19, chronic covid-19, and aspergilloma

^**^Other diagnoses include pulmonary hypertension, CTEPH, Wegner disease, silicosis, hydatid cyst, anthracosis, trauma, spontaneous pneumothorax, sarcoidosis, and no definite diagnosis

### All-cause mortality

Seventy-six (26%) patients expired of whom 17 (6%) were due to hemoptysis, and 59 (20%) were due to other reasons such as complications of the underlying disease (cancer, bronchiectasis, covid-19, infections). The mean survival time (95% CI) was 35.93 (33.6, 38.26) months for all-cause survival.

**Fig. 2:**
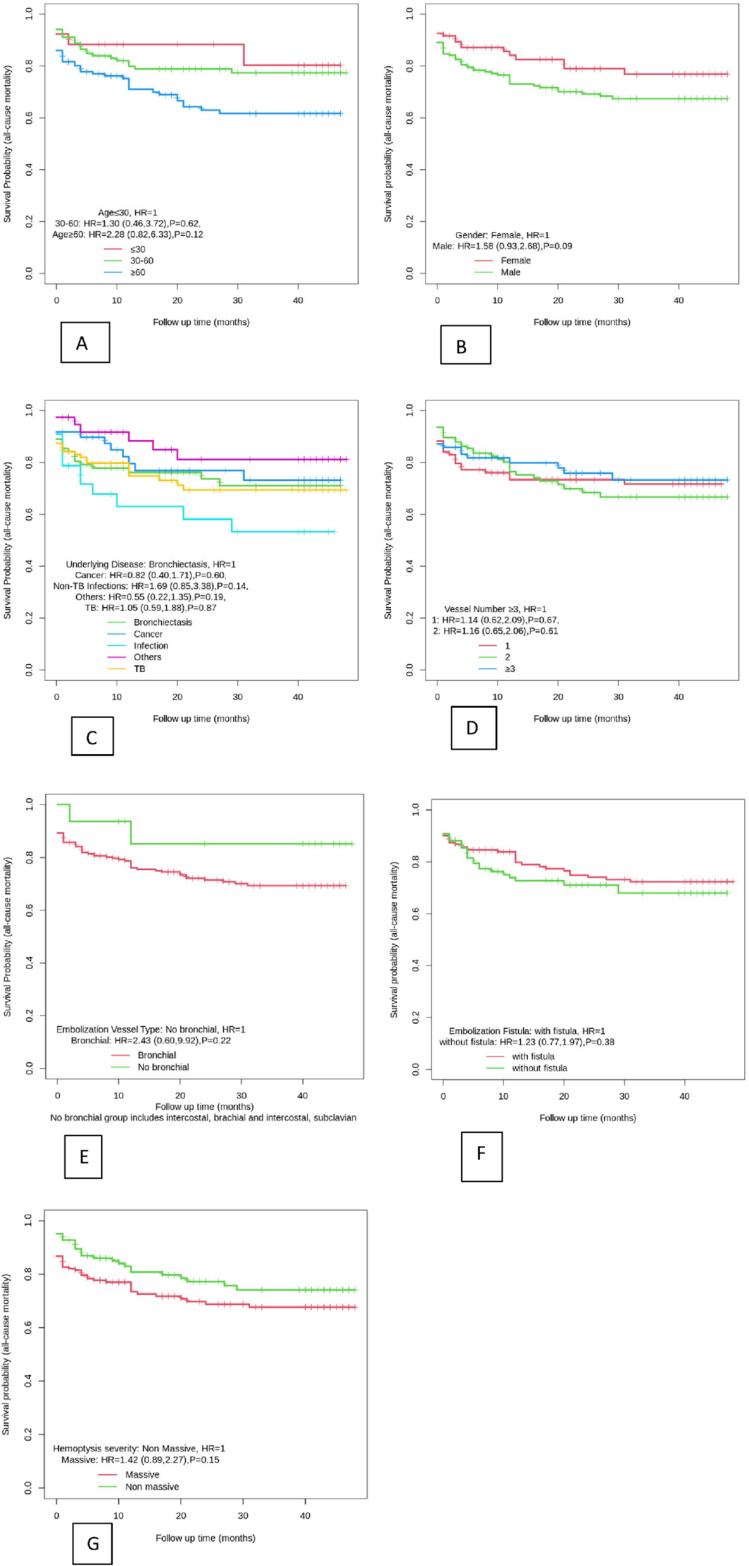
**A**, **B**, **C**, **D**, **E**, **F**, **G** KM curve and the HR (95% CI) for all-cause mortality for age, gender, underlying diseases, number of embolized vessels, embolized vessel type, fistula status, and hemoptysis status

Figure 2 shows the KM estimation and HR stating that mortality hazard for males was 58% higher than female mortality, which was not statistically significant [*HR* = 1.58, 95% *CI* (0.93–2.68), *P* = 0.09]. In addition, for patients aged 30–60 years, the hazard of death due to all causes was 30% more than the patients aged < 30 years old [*HR* = 1.3, 95% *CI* (0.46–3.72), *P* = 0.62]. The hazard of all-cause mortality was nonsignificantly higher in the patients aged > 60 years old in comparison to the patients aged < 30 years old [*HR* = 2.28, 95% *CI* (0.82–6.33), *P* = 0.12]. In massive hemoptysis patients, the odds of all-cause mortality were 42% (*OR* = 1.42, 95% *CI* 0.89 and 2.27) higher than in non-massive hemoptysis patients (*P* = 0.15).

The hazard of all-cause mortality was 14% and 16% higher in the patients with one and two involved vessels, respectively, compared to the ≥ 3 vessels [*HR* = 1.14 95% *CI* (0.62, 2.09), *P* = 0.67] [*HR* = 1.16, 95% *CI* (0.65–2.06), *P* = 0.61].

All-cause mortality hazard was almost 5% higher in old TB patients [*HR* 1.05, 95% *CI* (0.59–1.88), *P* = 0.87] and 69% higher in the patients with non-TB infections in comparison to the bronchiectasis cases [*HR* = 1.69, 95% *CI* (0.85–3.38), *P* = 0.14]. However, none of the relationships between underlying diseases and mortality hazard was statistically significant. Patients without arteriovenous fistula experienced an all-cause mortality risk of 23%, nonsignificantly higher than the patients with arterio-venous fistula (*P* = 0.38).

### Hemoptysis-related mortality

**Fig. 3:**
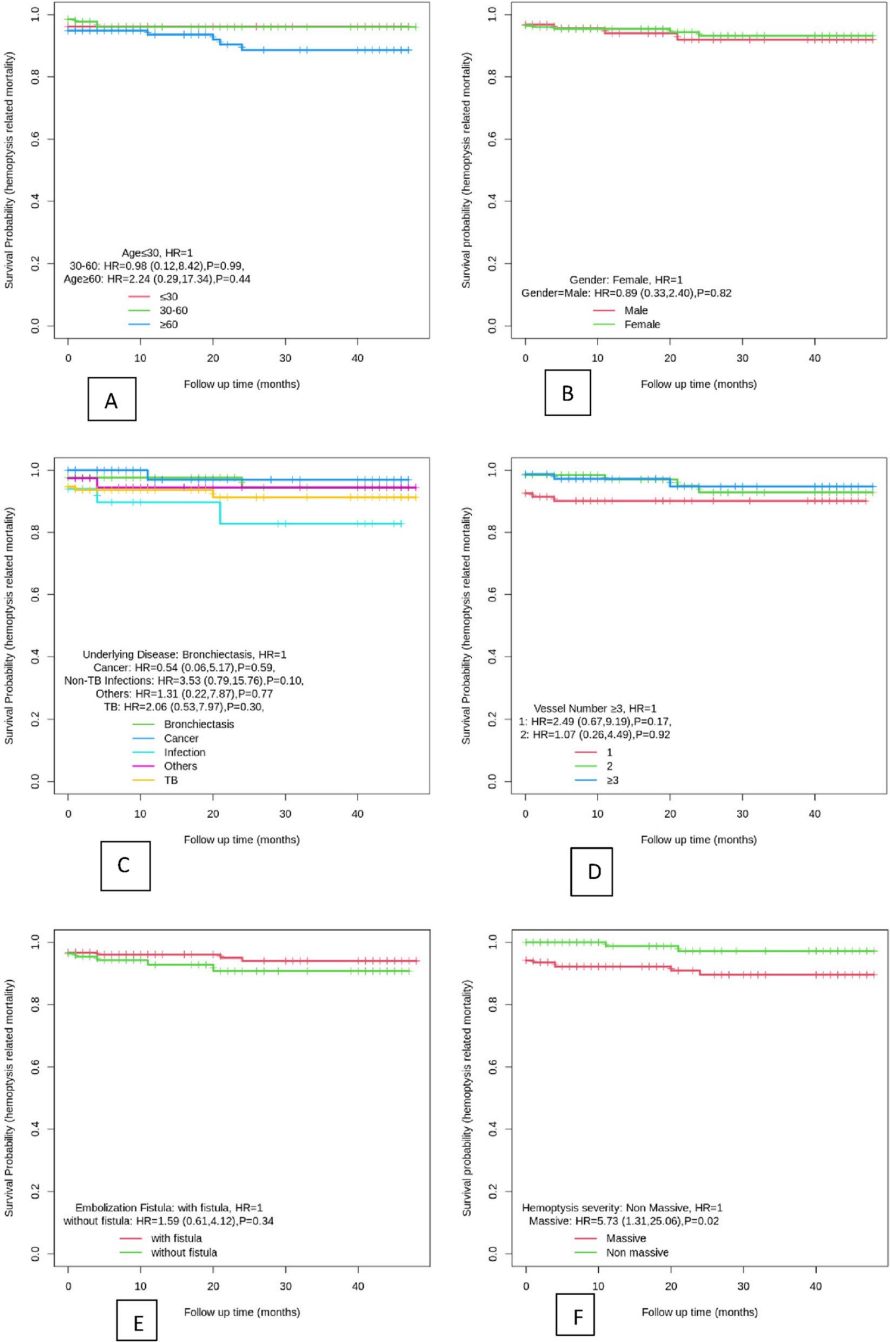
**A**, **B**, **C**, **D**, **E**, **F** KM curve and the HR (95% CI) for hemoptysis-related mortality for age, gender, underlying diseases, number of embolized vessels, embolized vessel type, fistula status, and hemoptysis status

The MST (95% CI) was 45.1 (43.75, 46.44) months for hemoptysis-related survival (Table 2). Figure 3 compares the KM curves for different levels of the studied factors. It was found that the hemoptysis-related mortality hazard for males was 11% lower than that of females (*P* = 0.82). Patients with old TB and non-TB infections experienced a mortality hazard of 2.06 and 3.53 times higher in comparison to bronchiectasis patients, respectively (*P* = 0.3, *P* = 0.1). In addition, patients without arteriovenous fistula had a higher mortality hazard than those with arteriovenous fistula (*HR* = 1.59%, 95 *CI* (0.61, 4.12), *P* = 0.34). Hemoptysis-related mortality was 5.73 (95% *CI* 1.31, 25.06) times in massive hemoptysis patients in comparison to the non-massive patients (*P* = 0.02).

**Table 2:** Survival and recurrence outcomes after bronchial artery embolization (*N*=297)

	Survival rates		Recurrence	
Time point	%Overall survival(all-cause)	%Hemoptysis-related survival(disease-specific)	*N* (%*)	%Recurrence
1 month	86.9	0.963	43 (21.6)	14.6
3 months	84.8	0.963	24 (12.06)	8.8
6 months	81.1	0.955	19 (9.54)	8
9 months	80.3	0.955	12 (6.03)	5.7
1 year	76.5	0.949	41 (20.6)	22.7
2 years	72.3	0.928	27 (13.56)	20.4
3 years	70.4	0.928	22 (11.05)	20.5
4 years	70.4	0.928	11 (5.52)	19.6
MST (95% CI)	35.93 (33.6, 38.26)	45.1 (43.75, 46.44)		

*MST* mean survival time in a month. *Recurrence rate was calculated as the number of patients who experienced recurrence divided by the number of patients still eligible for recurrence at each time point (“patients at risk”)

The hazards of hemoptysis-related mortality were 57% and 60% lower in the patients with two and ≥ 3 vessels involved compared to one vessel involved, respectively [*HR* = 0.43, 95% *CI* (0.14, 1.29), *P* = 0.13, and *HR* = 0.4, 95% *CI* (0.11, 1.48), *P* = 0.17].

The association between hemoptysis-related mortality and embolized vessels’ type was not assessed due to low sample size.

The PH assumption was satisfied for all Cox models. It is noteworthy that the results remained unchanged when the analysis was performed for the patients who survived at least 12 months after the procedure.

### Recurrence of hemoptysis

Table 2 summarizes the temporal pattern of hemoptysis recurrence, showing an early peak at 1 month after BAE (14.6%), a minimum recurrence rate at 9 months (5.7%), and a secondary rise to approximately 20% during long-term follow-up.

In the logistic regression analysis (Table 3), age showed a nonsignificant trend toward higher recurrence risk. Compared with patients aged ≤ 30 years, those aged 30–60 years had more than twice the odds of recurrence (*OR* = 2.23, 95% *CI* 0.87–5.71, *P* = 0.09), while patients aged ≥ 60 years also showed a modest but nonsignificant increase (*OR* = 1.35, 95% *CI* 0.52–3.49, *P* = 0.54). Male patients similarly demonstrated a non-significant 24% higher odds of recurrence compared with females (*OR* = 1.24, 95% *CI* 0.74–2.10, *P* = 0.42). Hemoptysis severity had a significant effect with patients with non-massive hemoptysis having 71% higher odds of recurrence than those with massive hemoptysis (*OR* = 1.71, 95% *CI* 1.05–2.80, *P* = 0.03), among whom 33.8% experienced at least one episode of recurrence.

**Table 3:** The association between the odds of at least one episode of recurrence and different factor using univariate logistic regression

Factor	Subgroup	At least one episode of recurrence (%)		OR (95% CI)	*P*
		No	Yes		
Age					
	≤ 30	18 (72)	7 (28)	1	
	30–60	68 (53.5)	59 (46.5)	2.23 (0.87, 5.71)	0.09
	≥ 60	80 (65.6)	42 (34.4)	1.35 (0.52, 3.49)	0.54
Gender					
	Female	57 (64)	32 (36)	1	
	Male	109 (58.9)	76 (41.1)	1.24 (0.74, 2.1)	0.42
Hemoptysis severity					
	Massive	104 (66.2)	53 (33.8)	1.00	
	Non-massive	63 (53.4)	55 (46.6)	1.71 (1.05, 2.8)	0.03
Underlying disease					
	Bronchiectasis	43 (57.3)	32 (42.7)	1	
	Cancer	33 (71.7)	13 (28.3)	0.53 (0.24, 1.16)	0.11
	Non-TB infections*	25 (78.1)	7 (21.9)	0.38 (0.15, 0.98)	0.045
	Old TB	46 (54.1)	39 (45.9)	1.14 (0.61, 2.13)	0.68
Embolization experience time					
	1	151 (65.9)	78 (34.1)	1	
	2	11 (44)	14 (56)	2.46 (1.07, 5.68)	0.03
	≥ 3	5 (23.8)	16 (76.2)	6.2 (2.19, 17.54)	< 0.001

*Non-TB infections include acute covid-19, chronic covid-19, and aspergilloma

Regarding underlying etiology, no statistically significant difference in recurrence odds was observed between patients with old tuberculosis and those with bronchiectasis (*OR* = 1.14, 95% *CI* 0.61–2.13, P = 0.68), whereas patients with non-TB infections showed significantly lower recurrence risk compared with bronchiectasis (*OR* = 0.38, 95% *CI* 0.15–0.98, *P* = 0.045). Repeated embolization was the strongest predictor of recurrence: patients undergoing two sessions had more than twice the odds (*OR* = 2.46, 95% *CI* 1.07–5.68, *P* = 0.03), and those with ≥ 3 sessions had over sixfold higher odds (*OR* = 6.20, 95% *CI* 2.19–17.54, *P* < 0.001) compared with single-session procedures.

Table 3 revealed that 157 of 173 (90.7%) patients with massive hemoptysis and 118 of 124 (95.1%) patients with non-massive hemoptysis survived 1 month after initial hemoptysis and bronchial artery embolization. Moreover, 108 patients experienced at least one episode of hemoptysis recurrence, of which 51% of recurrences were in patients with initial non-massive hemoptysis and 49% were in patients with massive hemoptysis. Nine patients (8.3% of those with recurrence) died from recurrent bleeding. There was a statistically significant association between recurrence, mortality, and hemoptysis severity (*P* < 0.05, Table 3S).

## Discussion

In this prospective cohort study, we evaluated BAE in 297 patients as a treatment for massive and non-massive hemoptysis caused by multiple etiologies, and the survival and recurrence rates were compared between these groups. Technical success, defined as immediate angiographic exclusion of the bleeding focus [11], was achieved in 91.1% of patients — consistent with previous reports of 70–99% [12].

More than half (58.2%) of the patients suffered from massive hemoptysis, and the most common etiology in both massive and non-massive hemoptysis was old TB (32%), followed by bronchiectasis (27.6%). This pattern aligns with prior studies where TB and bronchiectasis were also leading causes [13–15]; also, the percentage and order of these etiologies could be explained by the geographical epidemiology and the referral centers where the studies were conducted. Masih Daneshvari Hospital functions as the national tertiary referral center for pulmonary and respiratory diseases in Iran, which results in a higher case volume of patients with hemoptysis referred from different regions of the country. This referral pattern may explain the relatively large sample size observed in our single-center study.

Before proceeding with BAE, CT scans were performed to localize bleeding sites and disease extent. Bilateral abnormalities in the CT scan and bilateral bleeding vessels were found in 73 patients; right-side abnormalities in the CT scan and right-side bleeding vessels during BAE were found in 35 patients, while left-side abnormalities in the CT scan and BAE were found in 19 cases. These results demonstrate the compatibility of the side of embolized vessels in the BAE procedure and CT scan results for 20%. As such, a study conducted by Jung Han Hwan et al. demonstrated that contrast-enhanced CT scan examinations were common for diagnosis (95.3%) and could explain their high technical success rate (96.1%) [16]. Findings from the CT scan and the type of underlying disease— whether diffuse or local disease—could be used as a guide for the clinician to search for abnormal and bleeding vessels during the embolization procedure. Complications were rare (0.3% each for chest pain, limb ischemia, and spinal ischemia), comparable to reported rates of 0.19–6.5% [17].

In our 4-year follow up, the life expectancy of patients after BAE was approximately 3.5 years (35.92 months). Furthermore, the total mortality rate in this study was 26%, and only 5.7% were due to hemoptysis. In this regard, according to previous literature, approximately 5% to 14% of patients presenting with hemoptysis will have life-threatening hemoptysis, with a reported mortality rate between 93 and 8% [18]. In this study, the lowest survival rates were at the 3rd and 4th year after initial hemoptysis with 70.4%. Additionally, in a 5-year study conducted by Abdulmalak et al. in France, a high subsequent mortality rate of 21.6% and 27% at 1 and 3 years after hospitalization was reported [19]. Based on other literature, mortality rates in hemoptysis patients range from 4% at 1 year to around 20% at 2–3 years after initial referral for hemoptysis [20–22].

Recurrence rate was higher in patients with old TB with 45.9% of cases, followed by bronchiectasis with 42.7% which are also the most common etiologies of hemoptysis. In a retrospective study on 5793 patients of whom 43.6% underwent BAE and that was conducted by Onur et al., the most encountered causes in recurrence were lung cancer followed by bronchiectasis, TB sequela, and active TB [23].

In the current study, the recurrence rate in patients with arteriovenous fistula was 36.8%, and in patients with no evidence of fistula, it was 43%. However, in another study, arterio-artery or arteriovenous fistula was a risk factor for the recurrence of hemoptysis after BAE treatment [24].

According to literature, the hemoptysis recurrence rate after BAE ranges from 10 to 58%, with 0–29% experiencing early recurrence and 10–60% experiencing long-term recurrence [12, 25, 26]. In our study, 67% of patients had at least one episode of hemoptysis recurrence with the rates as follows: 1 month (21.61%), 3 months (12.06%), 6 months (9.54%), 9 months (6.03%), 1 year (20.6%), 2 years (13.56%), 3 years (11.05%), and 4 years (5.52%). So, in the patients who experienced recurrence, the 1-month and 1-year recurrence rates after the procedure were the highest with 21.61% and 20.6%, respectively. These percentages could indicate that the highest recurrence rate in the first month after embolization could be due to involved vessels not detected in BAE. Moreover, the second highest recurrence rate which is in the first year after BAE could be due to neoangiogenesis during this time. In this regard, a study conducted by Hayakawa et al. reported two peak times of recurrence: the first was 1–2 months after BAE and the second 12–24 months after the procedure[27]. In addition, reports on recurrence rates of hemoptysis are difficult to compare but vary considerably from 0.3% at 1 year to 16.3% at 3 years [28, 29].

Moreover, in this study, 8.3% of patients that experienced recurrence passed away due to hemoptysis. Furthermore, in these patients, the highest mortality rates were in old TB with 41.2% followed by non-TB infections with 23.5% (Table 4S). These findings could designate that patients should be monitored and followed precisely in order to decrease the mortality rates. Also, as reported in other studies with improvement of diagnostic and therapeutic approaches, the mortality rate in massive hemoptysis is ranged between 6.5 and 38% [30–33]. These findings could enhance the effect of BAE in reducing the mortality rate in time of recurrence.

Furthermore, in our study, around one-third of patients (33.8%) with massive hemoptysis and 46.6% of patients with non-massive hemoptysis experienced at least one episode of recurrence. This difference between massive and non-massive hemoptysis recurrence rates could indicate that the underlying disease and its severity could have an impact on the efficacy of BAE. At our center, BAE for non-massive hemoptysis is limited to patients with recurrent or persistent bleeding despite conservative therapy or those with high-risk features or identifiable vascular abnormalities. The higher recurrence in this group likely reflects chronic etiologies such as post-tuberculosis changes and bronchiectasis, as well as less extensive embolization compared with massive cases, which are treated more aggressively. Before BAE, all patients receive appropriate medical therapy and airway care, with BAE reserved for refractory or high-risk cases. Additionally, another finding in recurrence rates demonstrates that patients who have undergone BAE for one time have a recurrence rate of 34.1%, patients who have undergone BAE two times have a recurrence rate of 56%, and patients who have undergone this procedure three or more times have a recurrence rate of 76.2%. This result could fortify the effect of underlying disease on outcomes of BAE and also is in correlation to the findings of the systematic review which reported that the recurrence of hemoptysis after BAE could be resulted from incomplete embolization, recanalization of previously embolized arteries, or recruitment of new collaterals due to underlying disease progression [12]. Another limitation of our study is the heterogeneous nature of our cohort, which included patients with diverse etiologies such as infection, malignancy, and bronchiectasis, potentially influencing recurrence and survival outcomes. In the current study, hemoptysis-related mortality was 5.73 times higher in massive hemoptysis patients in comparison to patients with non-massive hemoptysis. Although these results are statistically significant, the wide confidence interval indicates that a larger statistical population in future studies could obtain a more explicit conclusion.

The strength of this study is evaluating BAE in both massive and non-massive hemoptysis and also multiple etiologies of hemoptysis for 4 years.

## Conclusion

BAE is an effective procedure to manage acute and life-threatening hemoptysis with low complication rates and results in low recurrence rates and improves patients’ survivals. However, the treatment of the underlying diseases could enhance the outcomes of BAE and decrease the recurrence rates.

## Supplementary Information

Supplementary Material 1.

## Data Availability

The data that support the findings of this study are available from the corresponding author upon request.
