# Supplementary material for: Efficacy of bronchial artery embolization in hemoptysis: longitudinal studyon survival and recurrence
Source: CVIR Endovasc. 2025 Dec 13;8:111. doi: 10.1186/s42155-025-00627-x (PMC12701884; doi:10.1186/s42155-025-00627-x)
Supplement: Supplementary file 1 — Supplementary Material 1. [file 42155_2025_627_MOESM1_ESM.docx]

| **Table 1S** Embolization and mortality description of the patients | | |
| --- | --- | --- |
| Factor | Subgroup | N (%) |
| Embolization Vessel type |  |  |
|  | Bronchial | 274(92) |
|  | Others^*^ | 23 (8) |
| Fistula |  |  |
|  | Yes | 183(62.5) |
|  | No | 110(37.5) |
| Embolization Zone |  |  |
|  | Bronchus | 105(42.9) |
|  | Upper bronchus | 59(24.1) |
|  | Lower bronchus | 17 (6.9) |
|  | Others | 64(26.1) |
| Embolized vessels Number |  |  |
|  | 1 | 94 (31.6) |
|  | 2 | 125 (42.1) |
|  | ≥3 | 78 (26.3) |
| Patients status |  |  |
|  | Expired (hemoptysis-related) | 17 (6) |
|  | Expired (Other causes) | 59 (20) |
|  | Alive | 221 (74) |
| Mortality status |  |  |
|  | Expired (Massive, hemoptysis -related) | 15 (88) |
|  | Expired (Non- Massive, hemoptysis -related) | 2 (12) |
|  | Expired (Massive, Other causes)  Expired (Non-Massive, Other causes) | 34 (58)  25(42) |
|  | Expired (Massive and non-massive, due to other causes) | 59 (100) |
| ^*^Others includes intercostal, bronchial-intercostal, and subclavian arteries | | |

| **Table 2S** The concordance between CT scan findings and side of embolization | | | | | |
| --- | --- | --- | --- | --- | --- |
|  | | Embolization vessel side | | |  |
| CT scan abnormality side |  | Bilateral | Right | Left | Total |
|  | Bilateral | 73 (53.3) | 29 (21.2) | 35 (25.5) | 137 |
|  | Right | 27 (40.9) | 35 (53.0) | 4 (6.1) | 66 |
|  | Left | 21 (51.2) | 1 (2.4) | 19 (46.3) | 41 |
|  | Total | 121 | 65 | 58 | 244 |
| The kappa measure of agreement was 0.213 (P<0.001) | | | | | |

| **Table** **3S** The association between recurrence and death and hemoptysis severity | | | | |
| --- | --- | --- | --- | --- |
| Event | Subgroup | At least one episode of recurrence  N (%) | | |
| Expire^*^ |  | No | | Yes |
|  | Hemoptysis-related | 1 (0.6) | 9 (8.3) | |
|  | Other causes | 37 (22.2) | 7 (6.5) | |
|  | Alive | 129 (77.2) | 92 (85.2) | |
| Hemoptysis severity^*^ |  |  |  | |
|  | Massive | 104 (62.3) | 53 (49) | |
|  | Non massive | 63 (37.7) | 55 (51) | |
| Total |  | 167 (100) | 108 (100) | |
| ^*^P<0.05 | | | | |

| **Table 4S** Mortality rates in patients with recurrence episodes categorized by the underlying diseases | | | | | | | |
| --- | --- | --- | --- | --- | --- | --- | --- |
|  | | Underlying disease | | | | | |
| Patients status |  | Bronchiectasis | Cancer | Non-TB Infections | Old TB | Others | Total |
|  | Expired (hemoptysis-related) | 3 (17.6) | 1 (5.9) | 4 (23.5) | 7 (41.2) | 2 (11.8) | 17 (100) |
|  | Expired (Other causes) | 18 (30.5) | 10 (16.9) | 9 (15.3) | 18 (30.5) | 4 (6.8) | 59 (100) |
|  | Alive | 61 (27.6) | 38 (17.2) | 20 (9.0) | 70 (31.7) | 32 (14.5) | 221 (100) |
|  | Total | 82 (27.6) | 49 (16.5) | 33 (11.1) | 95 (32.0) | 38 (12.8) | 297 (100) |
